# Notes and News

**Published:** 1888-08-04

**Authors:** 


### NOTES AND NEWS.

COLLECTED AND COMMUNICATED.

MR. W. H. BARCLAY has been appointed assistant surgeon to the Bristol Infirmary by a vote of 306, as against one of 14 recorded for Mr. R. R. Whishaw.

IN moving a vote of thanks to the honorary physicians and surgeons of the Sheffield Infirmary, the Rev. H. H. Wright said he thought the best thanks they could give the medical gentlemen would be to build them a new hospital. To this the medical gentlemen will no doubt add a hearty "Amen."

THE proposed inquiry into the management of the Leicester Provident Dispensary is still hanging fire. Is there no public-spirited citizen who, in the interests of all parties concerned, will make an effort to bring the business to a head?

MR. MARK WHITWELL suggests that an excellent way of signalising the visit of the Prince of Wales to Bristol will be the complete freeing of the Children's Hospital from debt The amount of the debt would not be a very heavy burden for the public, but is felt to be a serious weight by a single institution. The children of Bristol should be encouraged to make a public demonstration on behalf of their own hospital.

MR. E. T. CLIFFORD, London secretary to the Scottish Economic Life Assurance Society, has been appointed honorary manager to the National Pension Fund for Nurses. Mr. Clifford still retains his appointment with the Scottish Economic, and will guide the fortunes of the two institutions in future. It is mainly owing to the energy and tact of Mr. Clifford that the Scottish Economic has had such a markedly successful career in London, a career hitherto almost without precedent. The National Pension Fund is to be sincerely congratulated on this appointment, and there can be no doubt that the Fund will now receive an impetus and a permanent energy, which will make it, as it deserves to be made, one of the most successful institutions ever founded in connection with the philanthropic world.

So many polite people resort to Bath that it might have been expected that even the members of the Corporation would by this time have learnt the elements of decent behaviour. Not so, however! Mr. Day, who, according to a "Ratepayer" in the *Bath Chronicle*, is a well-known and respected engineer, has asserted that the wooden buildings of the statutory hospital are not properly protected. Mr. Day's letter is thereupon pronounced to be "impudent." One member of the Corporation declares him to be "a liar," and another an "infernal liar." This is really very encouraging to those who take a lively interest in the progress of the species. If Bath should be in existence in the year 10,000 it may be hoped that by that time even its Corporation will have entered the pale of civilisation.

SIMULTANEOUSLY with Mr. Clifford's appointment a new prospectus is being issued. This prospectus has been carefully prepared by the honorary manager, in conjunction with the consulting actuary, Mr. George King, F.F.A., F.I.A. It is really an explanatory pamphlet rather than a mere prospectus, and will clear up any doubts and difficulties which may remain in the minds of nurses as the result of recent attacks from interested quarters. Those attacks—thanks to Dr. Bristowe's admirable address, and to the clear and convincing statements in the *British Medical Journal*, and elsewhere—have completely collapsed, and the promoters of them are no doubt thoroughly ashamed of themselves. Hospital managers, matrons, and nurses will be supplied with copies of the prospectus on application. We have been favoured with a proof, and find it a most interesting little book which will well repay perusal.

THE quarterly report of the Oldham Infirmary makes mention of a new and valuable addition to the comfort of the institution. This is a hydraulic hoist, capable of receiving a stretcher with its occupant, and conveying it from floor to floor. The hoist can also be used for taking up food, fuel, etc., which will be a great saving of labour, as all these things have hitherto been carried upstairs. At the last quarterly meeting the governors had under consideration the need of additional bed-rooms for the nurses and the servants, as well as rooms required by the surgeons and the matron, and instructed the Visiting and Admission Committee to procure plans, and proceed with the work promptly. This has been done, and the contractors are making rapid progress with the erection. When finished there will be an addition to the house of twelve apartments, the want of which has been long felt. It is to be hoped that increased subscriptions will enable the committee to meet the cost of these improvements and the increased expenditure they entail.

THE following vacancies are announced this week, the amount of salary in each case being given after the name of the institution :—

**Resident Medical Officers.**

Belgrave Hospital for Children. Resident House Physician and Assistant Secretary.—£30 per annum, with board, etc.
Bristol General Hospital. Physician's Assistant.—£50 per annum, with board, etc.
East Suffolk and Ipswich Hospital. Assistant House Surgeon.—£20 per annum, with board, etc.
General Hospital, Nottingham. Senior Resident Medical Officer.—£120, with increase, board, etc.
London Temperance Hospital, Hampstead Road. Senior House Surgeon.—Fifty guineas per annum, with board, etc.
Royal Free Hospital, Gray's Inn Road. Senior Resident Medical Officer.—£104 per annum, with board, etc.

**Non-resident Medical Officers.**

Ital'an Hospital, Queen Square, Bloomsbury. Physician.
Parish of Kirkmichael, Banffshire. Medical Officer.—£95 per annum.
Rathdrum Union, Newcastle Dispensary. Medical Officer.—£110 per annum, and fees.

**Dispenser.**

St. Alban's Hospital and Dispensary.—£65 per annum.

---

ON July 18th, a public meeting was held at the Portman Rooms, Baker Street, to consider the increased usefulness lying before the Western Ophthalmic Hospital, which has just acquired the freehold of its premises. The chair was occupied by Sir Michael Hicks-Beach, and amongst others on the platform were Canon Barker, Canon Leigh, the Rev. J. R. Diggle, and Mr. Seager Hunt The proceedings were opened by the secretary, who read letters regretting their inability to attend from Lord Randolph Churchill, Baroness Burdett-Coutts, and others, and then gave a short account of the work of the hospital. During 32 years of beneficent activity this institution has developed from the mere dispensary of 1856 into a well-organized institution, with its efficient acting and consulting staff. It meets a real need, and is greatly appreciated by the many sufferers who have been ably treated within its walls, being the only eye hospital within reach of the fast-growing population to the north and west. The public support it has received has been very meagre, and unless the subscriptions are considerably increased in-patients cannot continue to be received, and the work must in that case be lamentably checked and cramped, to the discouragement of the surgical staff and the disappointment of the many sufferers to whom indoor treatment is absolutely necessary. Sir Michael Hicks-Beach spoke lengthily and well in the cause of the hospital, and his speech was listened to with eager attention by all present. He said that they were met to plead on behalf of a charity which existed solely for the treatment of diseases of the eye among the indigent poor, and it was therefore a special institution. It was a well-known fact that diseases of the eye were far more satisfactorily treated at special hospitals than others, for it often happened that in cases of disease of the eye that operations of great delicacy had to be performed, and serious complications were avoided and cures obtained which in a general hospital would be almost impossible. He need not, therefore, dwell upon the great necessity for such a hospital. There was, perhaps, nothing for which they should be so profoundly thankful as the blessing of sight. The generality of mankind were fortunately born with sight more or less good, which they retained until a later period of life, but anyone who had suffered, as they knew he had done, from failure of sight, in any degree, then began to appreciate what the blessings of sight were, and what a considerable influence any injury to the sight could exercise upon a man's power for good and his enjoyment of life. If this was true, so also was it true that much could be done by medical science to alleviate injuries of that kind, and he had himself to be most thankful for the benefits that medical science has conferred upon him. During the thirty-two years the hospital had been in existence, 54,000 persons had been treated, and 300,000 separate attendances had been made. On the motion of Canon Barker, seconded by the Rev. Mr. Ostley, a resolution was carried, which pledged all present to do their best to assist such a valuable institution. Mr. Seager Hunt moved a further resolution declaring it to be necessary to raise a special fund for the maintenance of the hospital. After a vote of thanks to the chairman, the meeting closed.

---

THE "Hospital Saturday" collection in aid of the Cottage Hospital, Beverley, made on Saturday, the 14th ult., realised £78 11s. 7½d., an increase of £26 over last year.

---

THERE was to be "no rest for the wicked" last Saturday, according to the *Eastbourne Gazette*, because of the large numbers of determined young ladies who were to attack "visitors and residents" unceasingly on behalf of the Hospital Saturday Fund. Does the *Gazette* wish it to be generally understood that the "visitors and residents" of Eastbourne belong exclusively to the "wicked" classes?

---

THE progress of the pay system of hospital relief and its popularity among the more self-respecting members of the working classes may be gauged from the report of the Metropolitan Provident Association, which now possesses an annual income from members' contributions of over £3,000. Branches have been established in almost every district in London, and most of these are self-supporting. As the local medical practitioners are invited to attach themselves to these branches, it is to be presumed that "the bitter cry of the general practitioner" is not heard in connexion with this association.

---

AN alarming outbreak of small-pox is reported from Manchester. All the cases, as yet, seem to have occurred in connection with one institution, the St. Joseph's Industrial School, a Roman Catholic charity. By special order of the Health Committee two batches of 46 and 26 girls, in all 72 cases, have been removed to the Infectious Diseases Hospital at Monsall. It is much to be desired that a careful and impartial inquiry will be made into the circumstances of each of these cases. The inquiry should specially determine how many of them have been vaccinated; what degree of thoroughness has characterised each vaccination, and also how far the severity of attack is modified by slight or efficient vaccination. There is an unusual opportunity for making an important contribution to the vaccination question, which it may be earnestly hoped will not be neglected or misused.

---

SOME striking and instructive information is furnished by the annual report for 1887, recently issued, of the Statistical Committee and Medical Superintendents of the Infectious Hospitals and Imbecile Asylums. These institutions necessarily employ a large number of nurses and other assistants, and it is a matter of public interest how far their health suffers from their constant contact with infectious diseases. Out of a total of 1,103 subordinate officials, as many as 240 suffered from some form of illness requiring ward treatment during the year, and the number of days they were "warded" was 4,896, or a little over 20 days each on an average. Yet, although so many were ill, and some of them for so protracted a period, the total number of deaths was only five. When it is borne in mind that these persons were in constant attendance upon fever cases of the most infectious and dangerous character, a death-rate of less than four per thousand among the nurses will show how comparatively slight the risks of attendants are when due precautions are observed. Of the deaths only two were from infectious fevers, viz., one from typhus and one from enteric. The other three were from diseases not peculiar to fever hospitals, one being phthisis, one from acute tuberculosis, and one from pneumonia.

